# Success of Maternal and Child Health Pipeline Training Programs: Alumni Survey Results

**DOI:** 10.1007/s10995-021-03370-6

**Published:** 2022-02-22

**Authors:** Priyanka Fernandes, Karina Wang, Jason Timmerman, Angela Reyes, Faye Holmes, Omonike A. Olaleye, Hamisu M. Salihu, Victoria A. Moerchen, Harolyn M. E. Belcher, Nikeea Copeland-Linder, Charlotte A. Noble, Cheryl A. Vamos, Anna Armstrong, Catrina R. Waters, Deneen Long-White, Claudia Brown, Madhavi M. Reddy, Alice Kuo

**Affiliations:** 1 University of California, Los Angeles, 911 Broxton Avenue, Los Angeles, CA 90024 USA; 2 Texas Southern University, 3100 Cleburne St, Houston, TX 77004 USA; 3 Baylor College of Medicine, 3701 Kirby Drive, Houston, TX 77098 USA; 4 University of Wisconsin-Milwaukee, 3409 N. Downer Ave, Milwaukee, WI 53211 USA; 5 Kennedy Krieger Institute, 707 North Broadway, Baltimore, MD 21205 USA; 6 University of Maryland, 1119 Taliaferro Hall, College Park, MD 20742 USA; 7 University of South Florida, 13201 Bruce B. Downs Blvd, Tampa, FL 33612 USA; 8 Alabama State University, 915 S. Jackson Street, Montgomery, AL 36104 USA; 9 Howard University, 2400 6th St NW, Washington, DC 20059 USA; 10 Health Resources and Services Administration, Maternal and Child Health Bureau, U.S. Department of Health and Human Services, 5600 Fishers Lane, Rockville, MD 20857 USA

**Keywords:** Maternal and child health, Pipeline training programs, Undergraduate students, Evaluation, Success, Maternal and child health bureau

## Abstract

**Introduction:**

The Maternal and Child Health (MCH) Pipeline Training Program, promotes development of a diverse health workforce by training undergraduate students from underrepresented minorities. We aimed to evaluate the success of this program based on three domains: (1) demographic characteristics, (2) academic and career development, and (3) attitudes towards the field of MCH and the training programs among graduates.

**Methods:**

Three domains of success were determined through a collaborative effort between current program directors and the funding agency project officers. The survey with questions related to the three domains was distributed via an online platform to graduates from seven sites (one former site and six current sites). Data were analyzed and presented utilizing descriptive statistics.

**Results:**

The survey was distributed to 550 graduates, 162 responded (37% response rate). Demographically, 78% were female, 54% were Black/African American, 22% were Latinx and 83% did not report any disability. Eighty percent of respondents applied to graduate/professional schools, 67% received admission. Graduates often continued to work in MCH fields (70%). Majority felt confident and knowledgeable in the field (89%) and agreed the faculty were supportive at their training sites (90%).

**Conclusion:**

The study highlights successes in recruiting from underrepresented minorities, particularly Black/African Americans and first-time college goers in the family into the MCH Pipeline Training Programs. Programs were successful in furthering academic and career development for most trainees. Attitudes towards MCH and the training programs were overwhelmingly positive. Continued support of these programs is critical in addressing health disparities and achieving health equity.

## Significance

*What is already known about the topic*? Maternal and Child Health (MCH) Pipeline Training Programs are an important part of creating a diverse and culturally well-represented workforce in the field.

*What the article adds to the literature*? Results from the largest evaluation study conducted on graduates from the undergraduate MCH pipeline programs funded through Health Resources and Services Administration’s Maternal and Child Health Bureau (MCHB), which highlight important areas of success and improvement.

## Introduction

Improving the health and well-being of women, children, infants, and families has been an ongoing national priority, as highlighted in Healthy People 2020 and 2030 (Browse Objectives—Healthy People 2030 | Health.Gov, 2020; Maternal, Infant, and Child Health | Healthy People 2020, 2020). While addressing the health of this population, it is important to appreciate the growing diversity in this country and the fact that underrepresented minorities (URM) are often disproportionately affected by poor health (Diversity and Health Equity in the Maternal and Child Health Workforce, 2016). Several research studies have shown better utilization of mental health services and higher quality of care reported when there is racial concordance between patients and their physician providers (Saha & Shipman, 2007). Therefore, there is an increasing recognition of the importance of increasing minority representation in the Maternal and Child Health (MCH) workforce.

While there have been some improvements to increasing diversity in health care professions, there is need for continued efforts in this area. In a recent report, different racial/ethnic groups were represented differently in the studied occupations, though Caucasians were overrepresented in 23 of the 30 health occupations (Sex, Race, and Ethnic Diversity of U.S. Health Occupations (2011–2015), 2017). In order to address the issue of diversity and inclusion in the MCH workforce, the Division of Maternal and Child Health Workforce Development (DMCHWD) of the Health Resources and Services Administration’s (HRSA) Maternal and Child Health Bureau (MCHB) has adopted a multifaceted approach (Diversity and Health Equity in the Maternal and Child Health Workforce, 2016). The MCH Pipeline Training Program is the only program through HRSA MCHB that directly invests in educating, mentoring, guiding, and training undergraduate students from economically and educationally disadvantaged backgrounds to increase their interest, retention and leadership in the MCH workforce (Maternal and Child Health (MCH) Leadership, Education, and Advancement in Undergraduate Pathways (LEAP) Training Program, 2020).

The program has funded 12 unique undergraduate programs since its inception in 2006 and has trained over 5000 undergraduate students across the country (Discretionary Grants Information System (DGIS), 2016). Over 71% of these students come from underrepresented racial groups; 29% of these students are Hispanic/Latino (MCH Pipeline Training Program Fact Sheet, 2019). In 2016, the program expanded the number of sites funded from four every funding cycle to the current six programs every funding cycle. Individual programs offer undergraduate students various activities such as academic and career advising, faculty and peer mentoring, research opportunities, specialized curriculum, community-based learning, and leadership seminars. While the structure and administration of the funded programs are varied and tailored to local needs and available resources, all programs align with the MCH Pipeline Training Program’s vision and goals of recruiting, training, and retaining students from historically underrepresented groups into maternal and child public health professions. Individual programs also follow common reporting requirements of the funding agency (e.g., metrics related to recruitment, diversity, placement into maternal and child health professions, etc.) which ensures consistency between programs.

In continuing to offer meaningful experiences to undergraduate students, the currently funded MCH Pipeline Training Programs in partnership with MCHB, aimed to evaluate the success of training programs to date through a survey distributed to graduates from all the programs.

## Methods

The study was a collaborative effort between Alabama State University, Baylor College of Medicine/Texas Southern University, Kennedy Krieger Institute, University of California, Los Angeles (UCLA), University of South Florida and University of Wisconsin-Milwaukee, and the project officers from MCHB. The program directors and the project officers from MCHB met on a monthly basis initially, and mutually agreed upon the domains of success for all training programs as it pertains to the larger vision and goals of MCHB of developing a diverse maternal and child health workforce which can successfully serve the needs of an increasingly diverse patient population (Diversity and Health Equity in the Maternal and Child Health Workforce, 2016). The domains of success took into account what has been previously published in the literature, individual program goals as it pertains to the local context, and the overarching goals of MCHB. Three main domains emerged after these meetings as described below. University of California, Los Angeles developed the survey based on these domains and improved it through an iterative process, based on recommendations received from the other sites and from MCHB. University of California, Los Angeles also served as the main coordinating site for this study in terms of (1) survey administration via an online platform, (2) ensuring multi-site Institutional Review Board (IRB) approvals, (3) completion of multi-site Data Use Agreements (DUA), and (4) final data analysis.

The centralized platform used for survey administration and data collection was Qualtrics. An anonymous link to the survey was included in an email, which described the study, outlined the voluntary nature of the study and obtained consent from participants. The email was distributed to the program directors of six programs and the program director of the seventh site, which was previously funded. Six of the seven program directors then distributed the emails to graduates of their programs, on whom they had valid email addresses. The last site conducted the survey internally using the information provided in the original email because of institutional limitations. Data from this site were de-identified, aggregated, and later shared with UCLA to include in the final analysis. The survey was administered to the various sites between June 2019 and March 2020.

The study was conducted in accord with prevailing ethical principles and the coordinating site ensured IRB approvals/exemptions and DUA were completed at all sites, as required by the parent institute of each individual training site.

The three domains of success evaluated included (1) demographic characteristics, (2) academic and career development among graduates, and (3) attitudes towards the field of MCH and the training programs among graduates. Within each domain there were multiple closed-ended questions asked including yes/no, multiple-choice (single and multiple answers), Likert-scale, and multiple open-ended questions. The individual program directors and funding agency project officers unanimously agreed that success in existing recruitment efforts and project activities are better evaluated by understanding the demographic breakdown of the students they trained. Demographic questions focused on obtaining background information including gender identity, race, ethnicity, disadvantages and disabilities. Academic and career development questions aimed at understanding whether graduates pursued additional degrees, their occupational status and the fields of their studies/occupations. Oftentimes in the workforce serving vulnerable communities, URM providers are disproportionately tasked with the care of populations facing multiple vulnerabilities (e.g., minorities, low socioeconomic status, etc.) (Saha & Shipman, 2007). The survey therefore allowed for respondents to choose multiple answers for questions pertaining to populations currently served. Questions on MCH attitudes focused on how graduates perceived the MCH Pipeline Training Program and the affiliated faculty, and their confidence in understanding the field at large. The survey instrument is included as a supplement.

Data from multiple sites were merged and analyzed. Descriptive data analysis of survey responses was completed, using frequency and percentage for categorical variables and mean and standard deviation (SD) for continuous variables.

## Results

The survey was distributed to 550 graduates from the MCH Pipeline Training Programs at seven sites (Table 1). The survey had a total of 162 responses, generating a 37% overall response rate. In general, the response rate was higher among the programs that were funded relatively recently compared to programs that have a longer funding period: three programs have been funded for 14 years, one program was funded for 10 years (currently not funded) and the remaining three programs have been funded for four years.

**Table 1:** Response rate

	Number of years funded	Number of graduates	Number of responses	Response rate (%)
Alabama State University	14	181	38	21
Baylor College of Medicine/Texas Southern University	4	40	8	20
Howard University	10	80	26	33
Kennedy Krieger Institute	4	12	6	50
University of California, Los Angeles	14	121	37	31
University of South Florida	4	11	7	64
University of Wisconsin-Milwaukee	14	105	29	27
Total		550	162	37

### Demographic Characteristics (Table 2)

**Table 2:** Demographic information

	Total (n = 162)	
	Mean	SD
Age in years	29	4
	Frequency	%
Gender identity		
Female	127	78
Male	27	17
Transgender female	0	0
Transgender male	0	0
Gender non-conforming	1	1
Race		
American Indian/Alaskan Native	1	1
Asian	21	13
Black/African American	87	54
Native Hawaiian/Pacific Islander	0	0
Caucasian	26	16
Multiple	6	4
Ethnicity		
Hispanic/Latinx	36	22
First in family to attend college	72	44
First-/Second-generation US citizen or permanent resident	80	49
Highest level of education in either parent		
Never went to school	1	1
Some school	35	22
High school diploma	18	11
Some college	23	14
College diploma	32	20
Graduate degree or higher	31	19
Household language other than English	67	41
Disadvantaged in the following ways (multiple choice)		
Was in foster care	1	1
Received free/reduced lunch at school	72	44
Received temporary assistance for needy families/other financial assistance benefits	18	11
Used food stamps regularly	31	19
Not disadvantaged	74	46
Other	10	6
Received following financial aid in college (multiple choice)		
Pell grant/state equivalent	103	64
Stafford loan/federal or state loan program	95	59
Work study	62	38
Private loan	31	19
Private scholarship or grant	76	47
No financial aid assistance	13	8
Disabilities (multiple choice)	18	11
Attention Deficit Hyperactivity Disorder	5	3
Autism	0	0
Learning disability	2	1
Deafness/Hearing impairment	1	1
Blindness/Visual impairment	1	1
Physical disability	2	1
Mental/Emotional disability	6	4
No disability	134	83

The mean age of responders was 29 years (SD 4). The majority identified as female (78%), and Black/African American (54%); about a fifth was Latinx. Caucasians (16%) and Asians (13%) were the next most represented groups in our sample. More than half (~ 60%) had parents who received less education than a college diploma and 44% of respondents were the first in their family to attend college. Most respondents (51%) weren’t US citizens or permanent residents and 41% spoke a language other than English at home. The majority reported being disadvantaged in one or more ways (54%). A large proportion of students received grants/scholarships as financial aid during college either through Pell grants (64%) and/or private scholarships/grants (47%). The vast majority (83%) did not identify as having any disabilities such as autism, a learning disability, and hearing/visual impairments.

### Academic and Career Development Responses (Table 3)

**Table 3:** Academic and career experiences

	Total (n = 162)	
	Frequency	%
Applied to graduate/professional school	130	80
Fields applied to (multiple choice)		
Public health	34	21
Medicine	23	14
Nursing	13	8
Social work	12	7
Education	6	4
Psychology	6	4
Physical therapy	8	5
Nutrition	4	2
Other^a^	44	27
Admitted to graduate or professional school	108	67
Completed graduate or professional school	63	39
Reason for non-completion of graduate/professional school (multiple choice)		
Could not afford	3	2
Could not handle academic work	1	1
Change in careers	1	1
Personal/family reasons	6	4
Currently in school	53	33
The Maternal and Child Health (MCH) Pipeline Training Program helped in application to graduate/professional school	78	48
Missing responses	82	51
The MCH Pipeline Program helped in being successful in graduate/professional school	77	48
Missing responses	83	51
Current work status		
Employed	100	62
Unemployed, full-time student	32	20
Unemployed, between jobs	2	1
Unemployed, other	6	4
Missing responses	22	14
Currently working with maternal and child health populations	114	70
Maternal and child health populations you currently work with (multiple choice)		
Pregnant women and mothers	31	19
Infants (< 1 year)	36	22
Toddlers and preschoolers (1–4 years)	34	21
School-age (5–12 years)	58	36
Adolescents and young adults (13–25 years)	55	34
Vulnerable populations you currently work with (multiple choice)		
Disability	63	39
Disadvantaged youth (foster youth, homeless)	64	40
Elderly	51	31
Low income	103	64
Medically vulnerable	64	40
Minority	105	65

^a^Includes Occupational therapy, Law, Dentistry, Speech and language pathology, and Audiology

Most graduates from the MCH Pipeline Training Program applied to graduate or professional schools (80%) and 67% were accepted into these programs. Public health (21%), Medicine (14%) and Nursing (8%) were the most common health-related fields to which survey respondents applied. Only a very small percentage did not complete graduate/professional school after being admitted because of affordability (2%), inability to handle the academic load (1%), and personal or family reasons (4%). The MCH Pipeline Training Program helped 48% in applying to (98% of question respondents) and 48% be successful in (99% of question respondents) graduate/professional school.

Among survey respondents, 62% were employed either part-time or full-time. Most continued to be involved with MCH populations (70%), with more than a third working with school-aged (36%) and/or adolescents and young adults (34%). Graduates from the program reported working with vulnerable minority (69%) and low-income (64%) populations.

### Attitudes towards the field of MCH (Figs. 1 and 2)

**Fig. 1:**
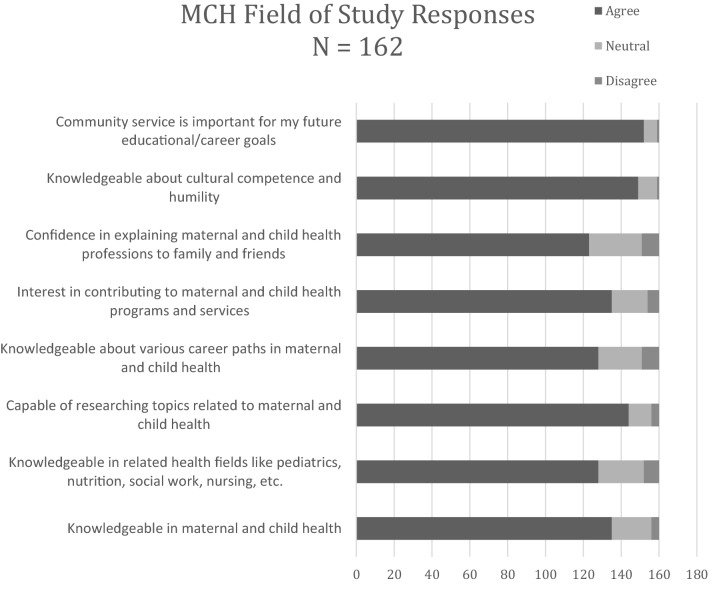
top. Responses to maternal and child health (MCH) as a field of study

**Fig. 2:**
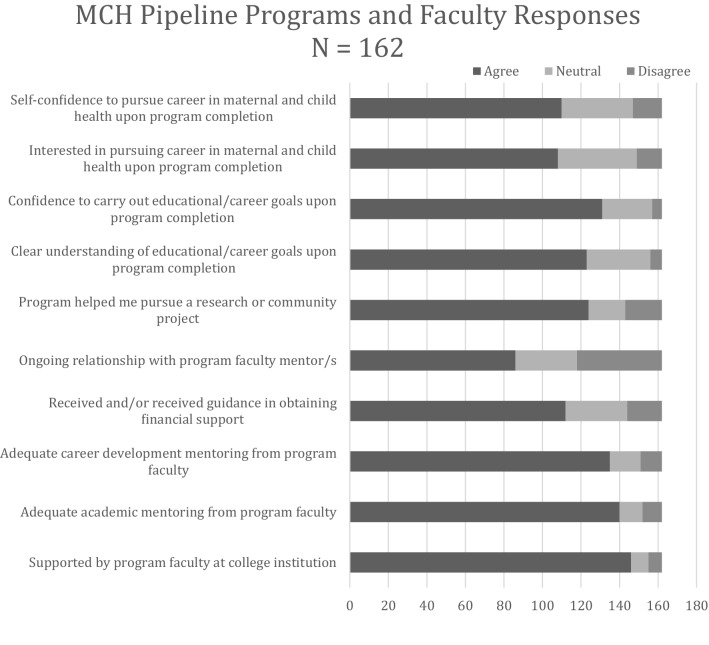
top. Responses to maternal and child health (MCH) pipeline programs and faculty

Graduates overwhelmingly felt a general sense of confidence and knowledge as it related to MCH issues and careers (Fig. 1): 89% felt capable researching MCH issues, 92% felt knowledgeable about cultural competence and humility, and 94% agreed that community service played an important part in their educational/career goals. When it came to the MCH Pipeline Training Programs, 90% felt supported by their faculty at their sites, 86% felt they received adequate academic mentoring and 83% felt they received adequate career development mentoring (Fig. 2). A lower proportion (53%) were able to continue an ongoing relationship with the program faculty mentor.

## Discussion

The current study is the largest evaluation conducted to date on the MCHB-funded MCH Pipeline Training Programs since its initial funding period in 2006. The strength of the study lies in the fact that responses were obtained from graduates from all funded sites, including past and present. The created survey was a collaborative effort between the currently funded programs and the funding agency, which allowed for common domains of success to emerge and be studied.

Published data on the demographic information of students enrolled and graduating from the undergraduate pipeline training programs are limited (Guerrero et al., 2015; Kuo et al., 2015; MCH Pipeline Training Program Fact Sheet, 2019). Our study adds relevant information in this regard and shows a good proportion of Black/African American and fair proportion of Asian representation among students recruited into and graduating from the MCH Pipeline Programs across the country. Recruitment and graduation of students from indigenous backgrounds and Latinx communities can be improved. Our sample had little representation from disabled populations among the respondents. However, the programs were successful in identifying and mentoring students who were the first in their family to attend college.

When it came to academic and career development, the training programs did very well in mentoring students to pursue higher education through graduate or professional schools. Our data is comparable to previous data from a single site in terms of the most popular graduate degrees pursued by alumni (Kuo et al., 2015). While the health professions chosen by graduates were quite varied (ranging from Public Health to Physical Therapy), fields such as Law, Dentistry and Speech/Language Pathology could benefit from better representation. The undergraduate pipeline training programs were also successful in having most graduates continue to work in the MCH field and with various vulnerable populations, similar to other MCHB-funded graduate-level pipeline training programs (Kavanagh et al., 2015).

In general, graduates from our program had overwhelmingly positive attitudes toward the MCH field, which has previously been shown in data from a single undergraduate MCH Pipeline Training Program site (Guerrero et al., 2015). They felt confident and knowledgeable about the field, careers in the field, and cultural competence and humility. The ability to participate in community service was valued highly among graduates in planning their careers. The training programs were successful in providing academic and career development mentoring and experiences to trainees as evidenced by the reported confidence in and understanding of educational/career goals upon program completion. An area for improvement includes increasing the involvement of graduates from the various programs. Some ideas include creating an alumni network and creating a mentoring program with graduates from the program.

The study had a few limitations. The training programs offer short-term, medium-term and long-term training based on the number of hours spent in the training program and the current study does not capture the differences between the different types of trainees. Details on Ethnicity were not elicited through the survey, limiting the generalizability of reported success within this heterogenous group. The response rate was lower than anticipated because email information on graduates from earlier years was either not systematically collected or outdated. The differences in response rates between programs also did not allow for robust between-site data analyses. Delays related to receiving IRB approvals/exemptions and DUA could have accounted for some of the variability in responses between sites.

## Conclusion

The results from our evaluation study highlight the successes of the MCHB funded MCH Pipeline Training Programs in three domains. Programs succeeded in recruiting from underrepresented minorities, particularly Black/African Americans and first-time college goers in the family. They were successful in furthering academic and career development for most trainees. Attitudes towards the field and the training programs were overwhelmingly positive. Areas of improvement include better representation through purposeful recruiting, increasing experiences in lesser-known MCH-related health professions and increasing graduate involvement in the programs. Continued and improved evaluation of training programs to include surveys of current trainees, graduates, and program directors and faculty, is important for better tracking of program successes and areas of need and will help identify between-site and between-group trends.

## Data Availability

Not applicable.
